# Integrating Non-surgical Endodontic Therapy With Vital Pulp Therapy as a Promising Approach: A Case Report

**DOI:** 10.7759/cureus.81637

**Published:** 2025-04-03

**Authors:** Kaadhambari Sivaguru, Vinaykumar G, Vinoo Subramaniam Ramachandran, Sibi Swamy, Abinaya K

**Affiliations:** 1 Conservative Dentistry and Endodontics, RVS Dental College and Hospital, Coimbatore, IND

**Keywords:** combination approach, endovital therapy, minimally invasive approach, non-surgical endodontic therapy and pulpotomy, symptomatic irreversible pulpitis and apical periodontitis, vital pulp therapy

## Abstract

Teeth with apical periodontitis and symptomatic irreversible pulpitis are often treated with root canal therapy, which is more invasive. However, vital pulp therapy enables the preservation of radicular pulp following the removal of deteriorated pulp in cases of irreversible pulpitis. With an emphasis on a minimally invasive approach, the case reported here has been treated by integrating non-surgical endodontic therapy with vital pulp therapy. Combining these two methods treats the infection and inflammation while preserving the tooth's vitality, which might result in better treatment outcomes and long-term success.

## Introduction

The vitality of the tooth is ascribed to the dental pulp, a highly vascularized and innervated tissue. Dentinogenesis is initiated by the odontoblast layer situated at the periphery of the pulp, resulting in the formation of primary dentin [1]. Pulpitis is the inflammation of pulpal tissue induced by microbiological, chemical, or physical (mechanical and thermal) irritants. Pulpitis is histologically categorized as acute, chronic, or hyperplastic, while clinically it is designated as reversible or irreversible [2]. If the inflammatory process advances from the pulp space to the periapical region due to persistent elevations in pulp tissue pressure, then it renders the tooth non-vital. Depending on the progression of inflammation, the tooth can either be partially or entirely non-vital. Necrobiosis is the term Grossman used to describe a tooth that is partly necrotic but still has an inflammatory pulp. A tooth with many canals may have necrosis in one canal and inflammation in others, or it may have a necrotic coronal and inflammatory apical pulp [3]. When the inflammation progresses to the periapical region, it results in apical periodontitis.

Pulpitis is considered reversible when it resolves by conservative treatment, such as restoration [4]. A complete pulpectomy is the definitive treatment for irreversible pulpitis (IP), a condition wherein the pulp is incapable of recovery [4]. A precise diagnosis of pulp condition is vital in selecting the optimal course of action and achieving a favorable outcome [5].

Root canal treatment is the standard option for teeth with apical periodontitis with symptomatic IP. And in worst cases, the extraction of a tooth is advised. These approaches can effectively treat the disease but are invasive, time-consuming, and costly. Thus, non-invasive or minimally invasive approaches ought to be the chosen therapeutic modality [6].

Histological examination of carious pulp exposure may reveal both injured and healthy pulp at different levels within the pulp canal area since the spread of inflammation in pulp takes place in compartments [4]. This confirms that in IP, inflammation is present only in the region adjacent to the carious exposure, and it will not spread to areas more than 2 mm from the exposure site [7]. So, vital pulp therapy would be the optimal treatment option with a success rate of 98.3% substantiating this viewpoint [8].

In vital pulp therapy, the radicular pulp may be conserved after the amputation of pulp exhibiting degeneration and irreversible changes [4,9]. It advocates for minimally invasive endodontics, emphasizing the preservation of the tooth's form and function.

This has resulted in integrating non-surgical endodontic treatment with vital pulp therapy preserving tooth vitality and facilitating healing while tackling the infection and inflammation linked to these diseases. This also enhances the long-term prognosis for patients with symptomatic IP and apical periodontitis [10,11].

This case report presents a patient who had IP with symptomatic apical periodontitis treated with the integration of vital pulp therapy in the distal canal and non-surgical endodontic therapy in mesial canals.

## Case presentation

**Figure 1:**
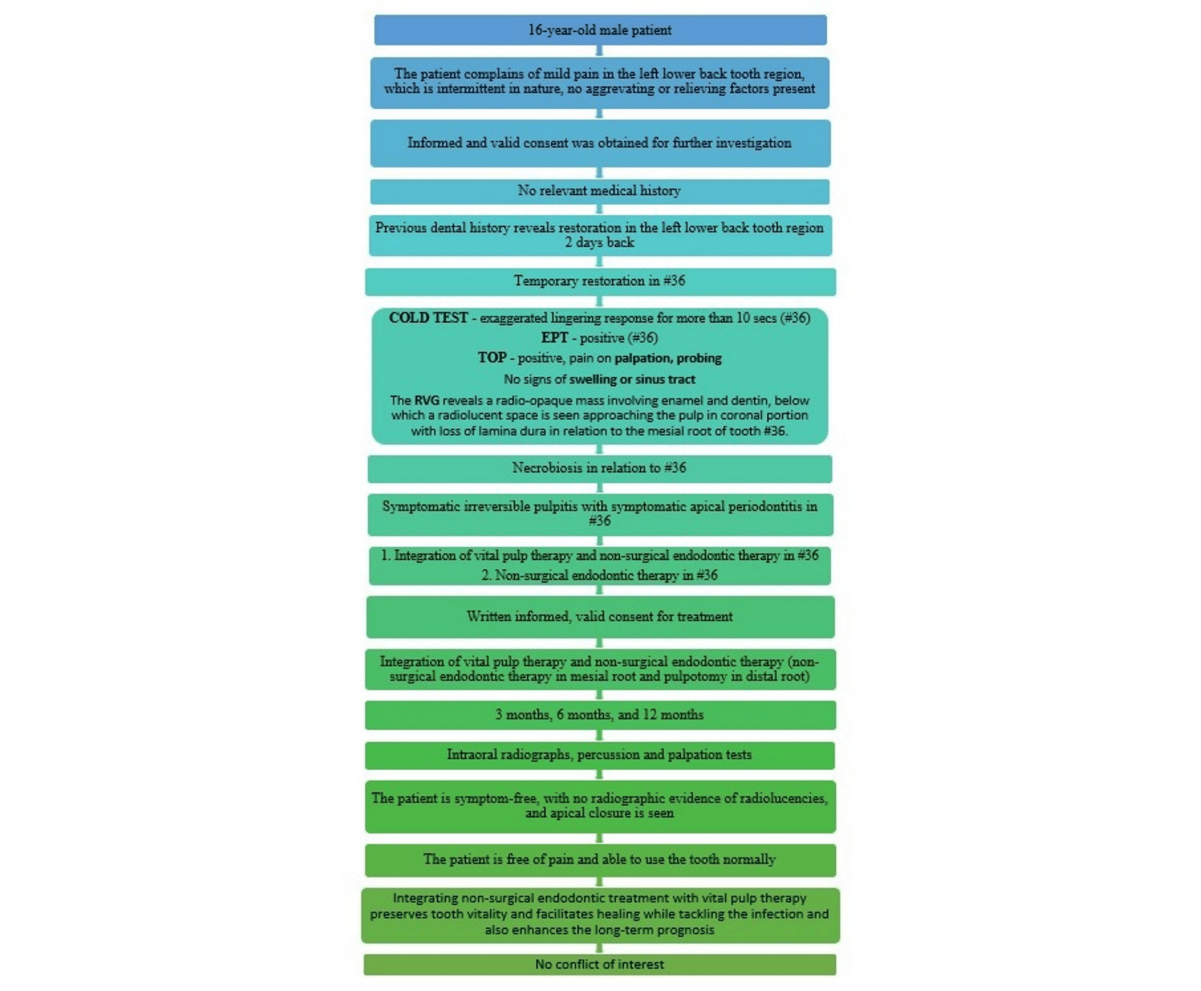
Case report in accordance with the 2020 PRICE criteria PRICE: Preferred Reporting Items for Case Reports in Endodontics; EPT: electric pulp test; RVG: RadioVisioGraphy

This case report has been composed in accordance with the 2020 Preferred Reporting Items for Case Reports in Endodontics (PRICE) criteria (Figure 1) [12].

**Figure 2:**
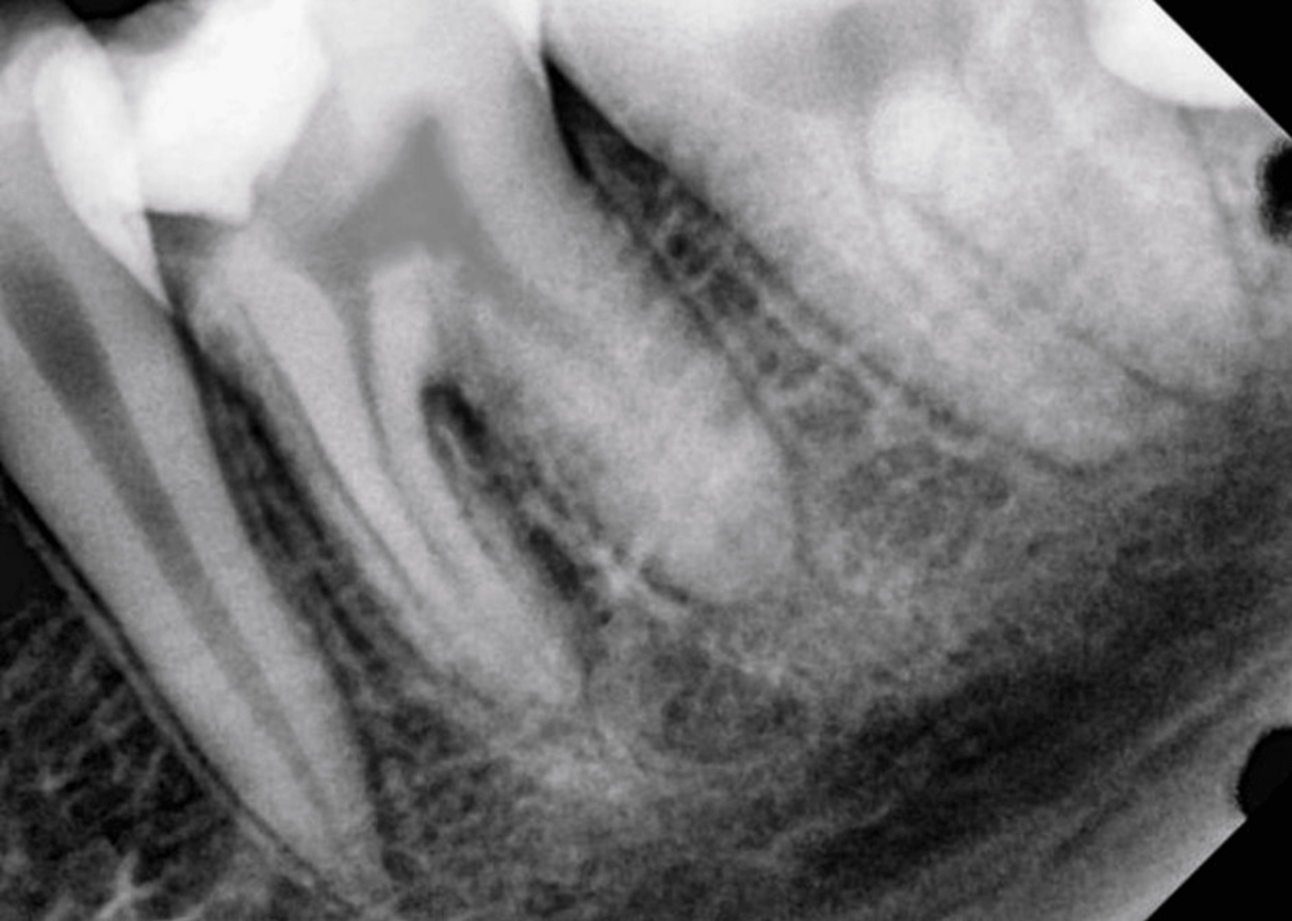
Periapical radiograph of tooth #36 with a temporary restoration

A 16-year-old male patient reported to the Department of Conservative Dentistry and Endodontics with a complaint of dull pain in the left lower back tooth region for the past one week, which is intermittent in nature. The past dental history revealed that the patient had a temporary filling in the tooth two days back in a nearby private clinic following which pain did not subside. On clinical examination, temporary restoration was present in tooth #36. The patient reported pain on palpation, probing, and percussion but showed no signs of swelling or sinus tract. The electric pulp test (EPT; Kerr Vitality Scanner, SybronEndo, Glendora, CA, USA) on tooth #36 was positive, and the thermal (cold) test (Green Endo-Ice®, Coltene Whaledent, Cuyahoga Falls, OH, USA) resulted in lingering pain longer than 10 seconds. The periapical radiograph showed a radio-opaque mass involving enamel and dentin, below which a radiolucent space is seen approaching the pulp in the coronal portion with loss of lamina dura in relation to the mesial root of tooth #36 (Figure 2). Based on the findings, tooth #36 had been diagnosed with symptomatic IP with symptomatic apical periodontitis. The treatment options like non-surgical endodontic treatment, vital pulp therapy, or a combination were explained to the patient.

**Figure 3:**
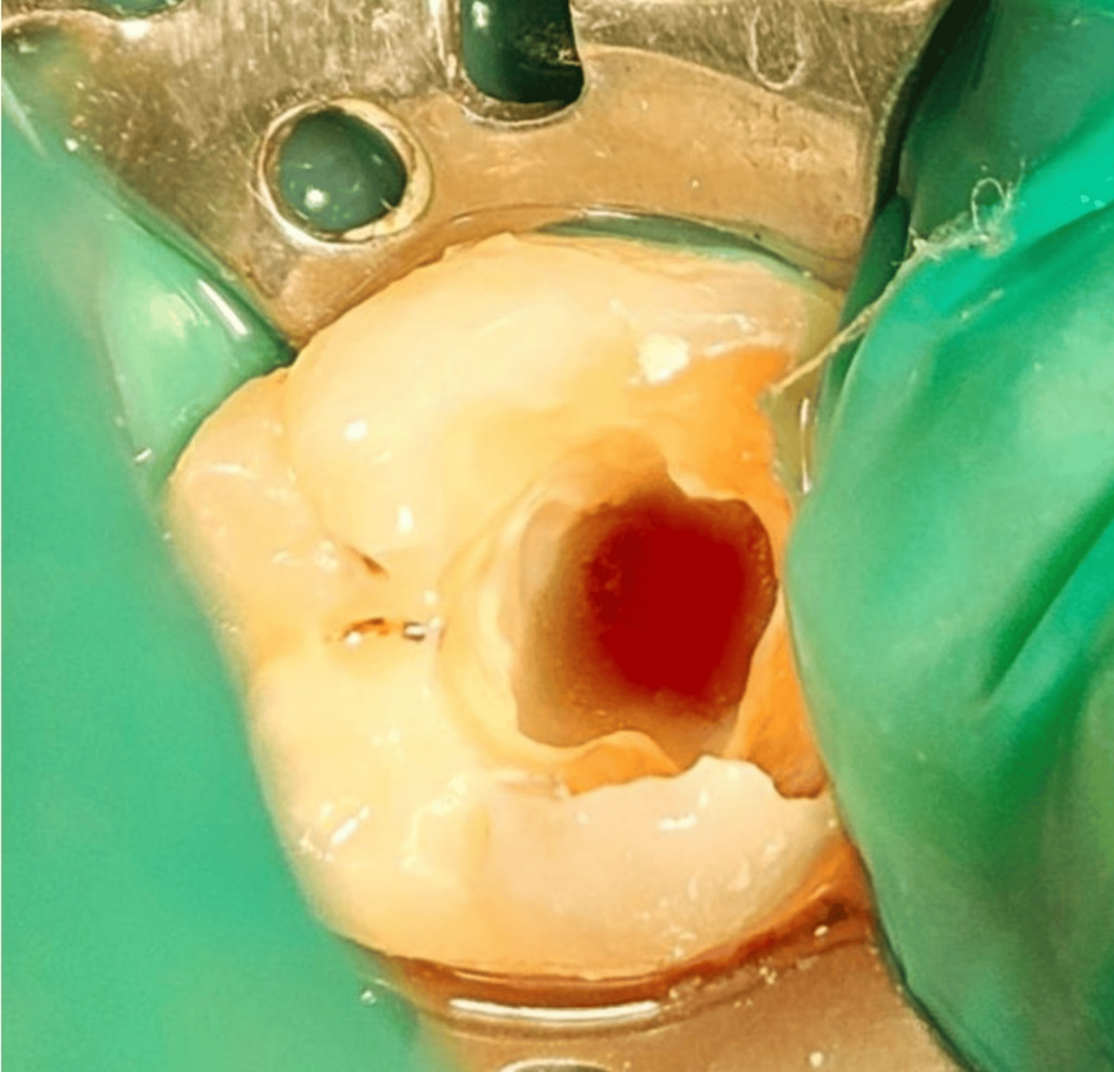
Profuse bleeding from the distal canal

The local anesthesia was achieved with lidocaine 2% with adrenaline 1:100,000 (Lignox 2%, Indoco Warren) followed by rubber-dam isolation (Hygenic, Coltene Whaledent, Cuyahoga Falls, OH, USA). The caries were excavated with a high-speed airotor handpiece and round bur. On access opening, the mesial orifices remained necrotic while bleeding persisted in the distal canal orifice (Figure 3).

**Figure 4:**
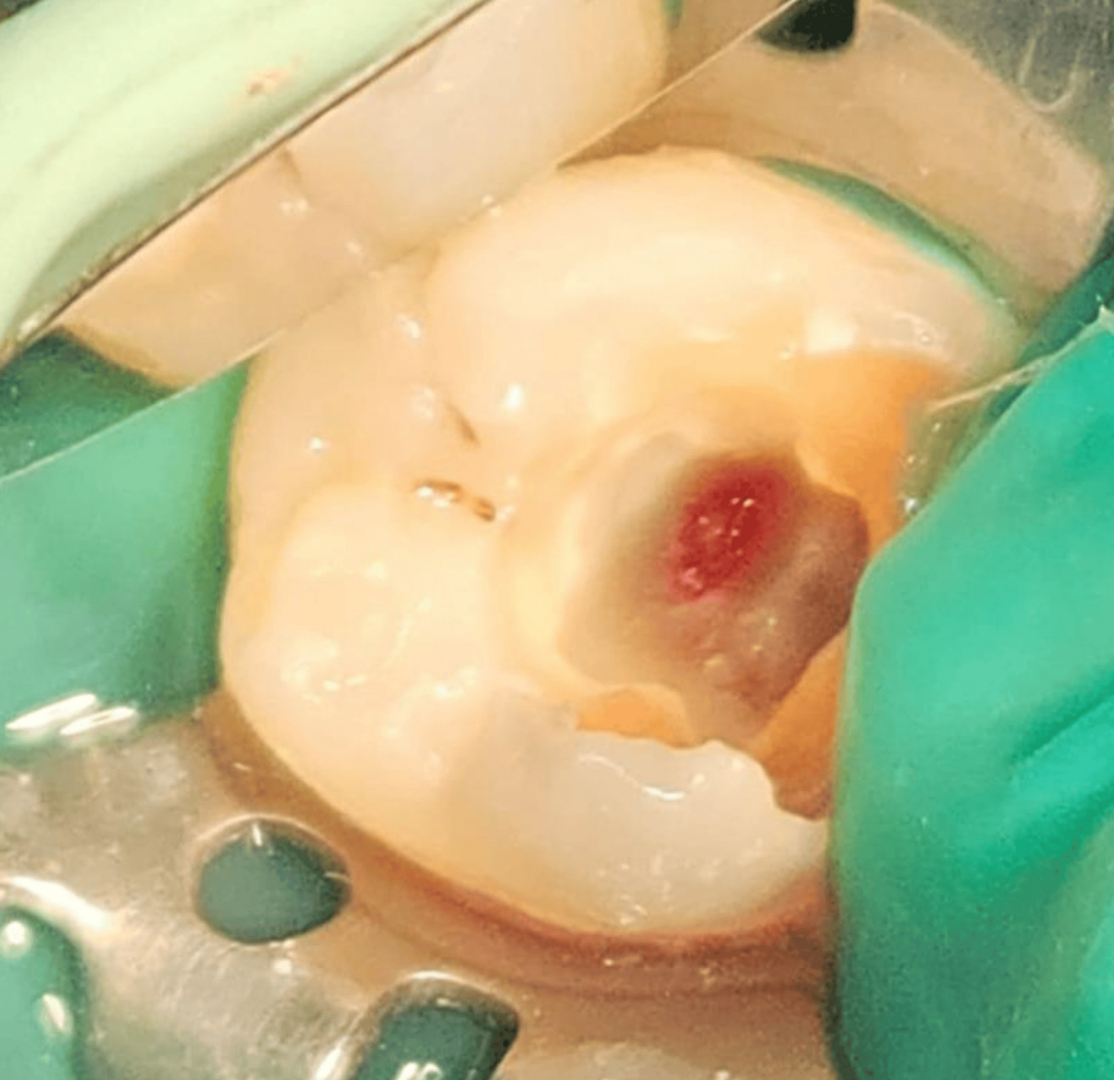
Arrest of bleeding from the distal canal

**Figure 5:**
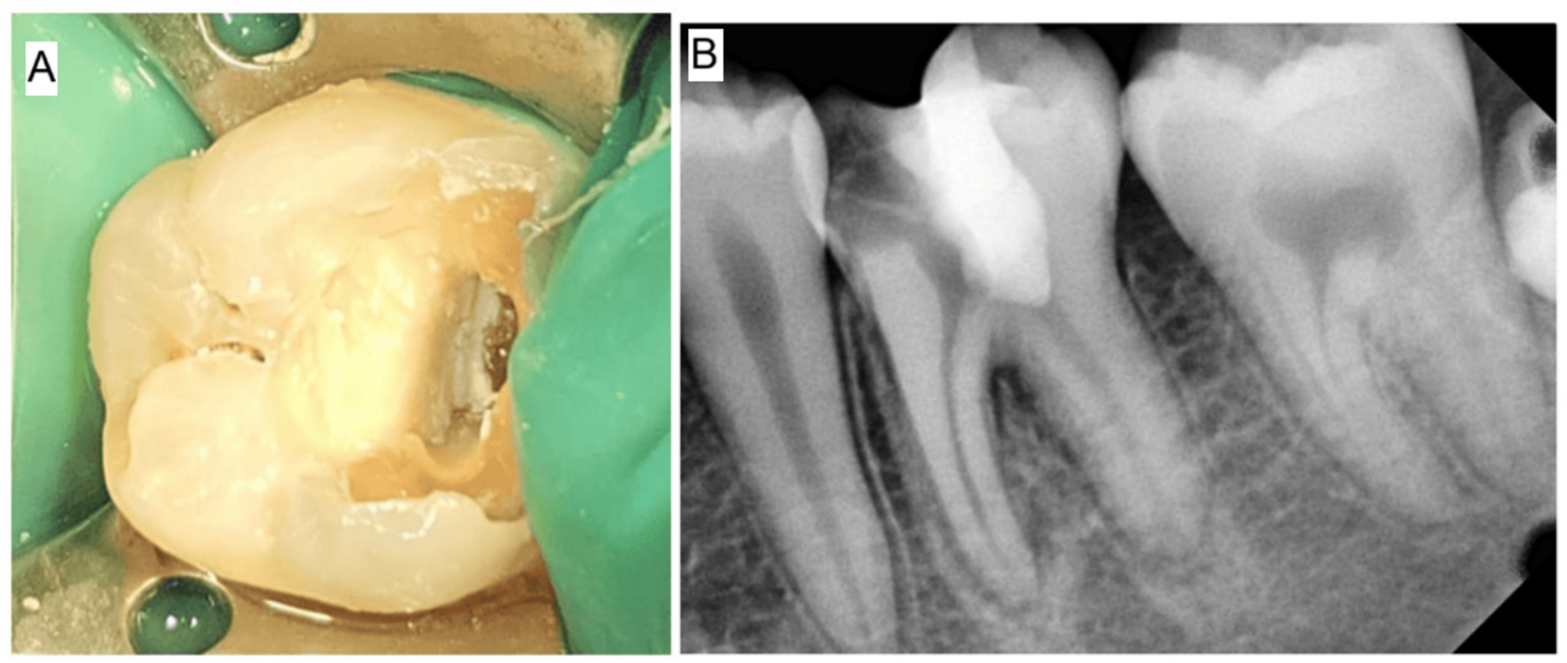
(A, B) Placement of MTA with RMGIC barrier in the distal canal MTA: mineral trioxide aggregate; RMGIC: resin-modified glass ionomer cement

A high-speed diamond bur was used to remove the exposed pulpal tissue to the level of the distal canal orifice. By using a 5% NaOCl moistened cotton, followed by a pressure pack with dry cotton, the hemostasis was established within three minutes [6]. Once the bleeding was controlled (Figure 4), mineral trioxide aggregate (MTA) (Angelus) of 2-3 mm thickness was packed with a condenser above the distal orifice. After 15 minutes over the set MTA, a layer of light-cured resin-modified glass ionomer cement (RMGIC) (Fuji II LC) was applied and 20 seconds of light curing was done (Figure 5).

**Figure 6:**
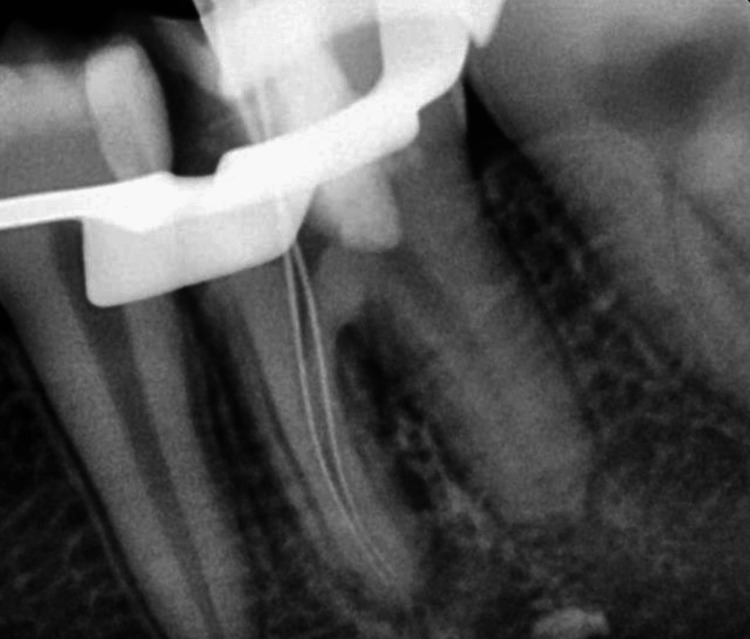
Working length determination in mesial canals

A non-surgical endodontic procedure was performed in the mesial canals after the completion of pulpotomy in the distal canal. Working length determination (Root ZX, J Morita USA, Inc., Irvine, CA, USA) was done (Figure 6), hand instrumentation was done till the #15 K file, apical preparation was done till 25.6% using Coltene Hyflex CM files, and it was accompanied by irrigation with 5 mL 5.25% NaOCl (Prime Dental Product Private Ltd, India) after each instrumentation cycle.

**Figure 7:**
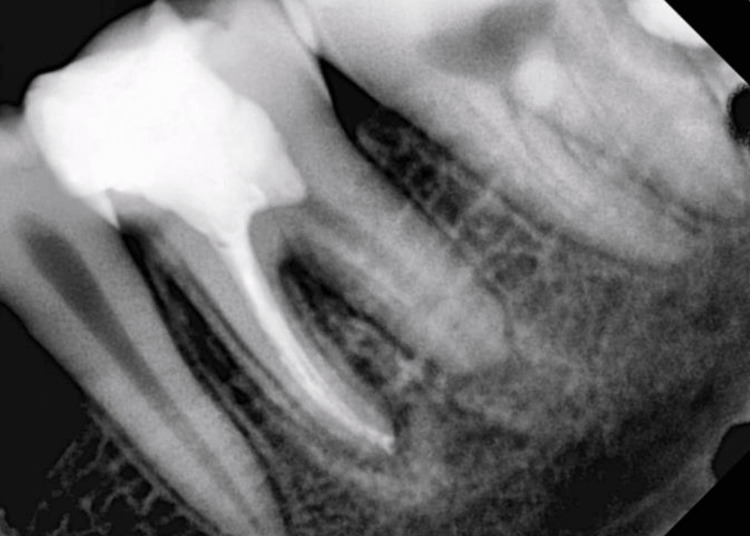
Post-operative radiograph

After enlarging, the canals were irrigated with 5 mL of 17% ethylenediaminetetraacetic acid (EDTA) for one minute. After drying with sterile absorbent points, the canals were filled with premixed calcium hydroxide paste using a Lentulo spiral. The tooth was then temporarily restored with the double-seal technique, in which Cavit was placed inside the pulp chamber, followed by glass ionomer cement (GIC) to cover the access cavity. The patient was recalled after one week. At the next appointment, the paste was removed with the help of XP Endo Finisher and copious irrigation with 5.25% NaOCl followed by a final rinse of 5.0 mL 17% EDTA, and then, the canals were obturated with gutta-percha and bioceramic sealer (CeraSeal, Meta Biomed, Europe). The access cavity was sealed with direct composite restoration (Tetric N-Ceram, Ivoclar Vivadent, Schaan, Liechtenstein) (Figure 7).

**Figure 8:**
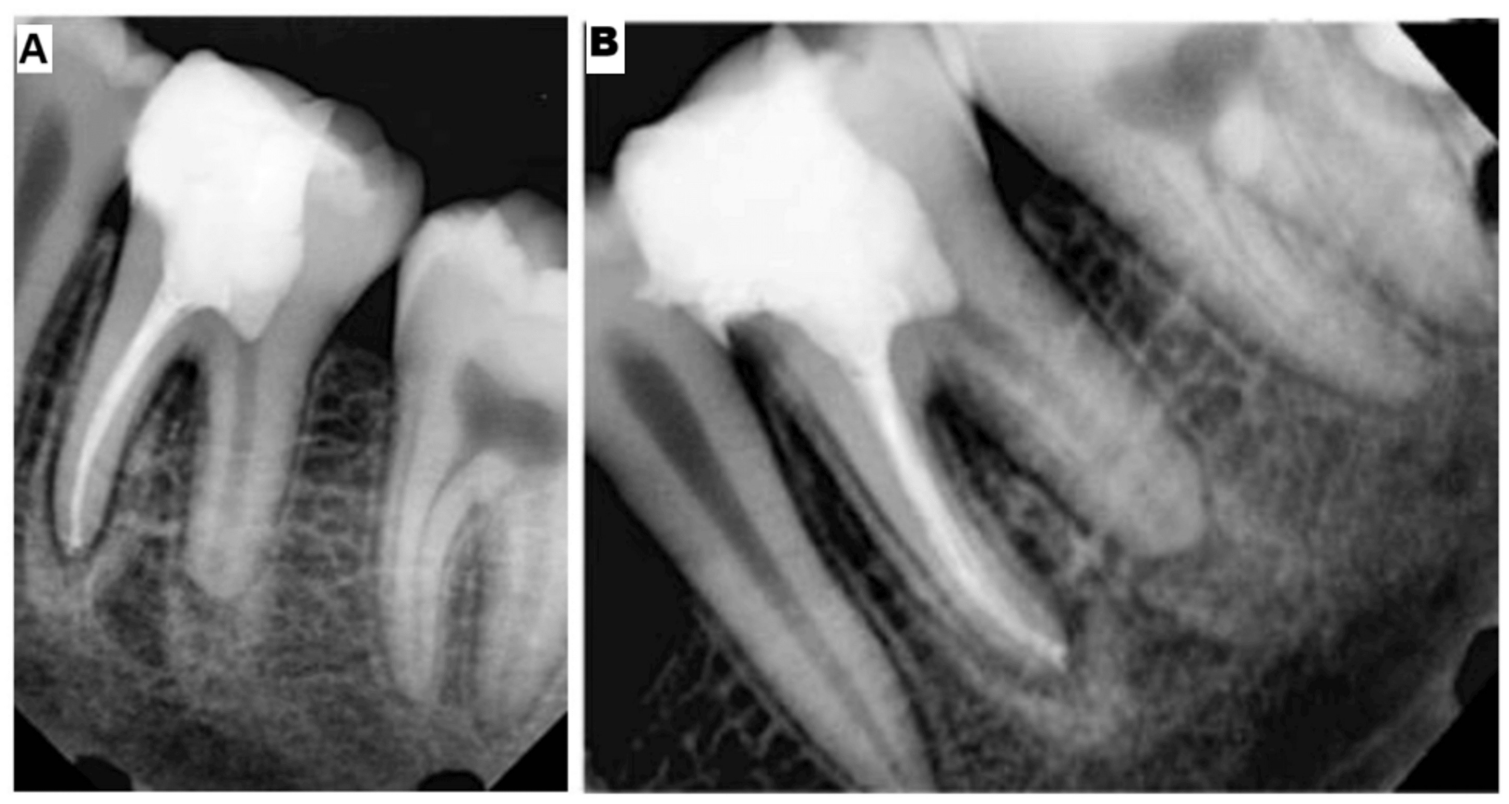
(A, B) Follow-up radiograph after one year

The patient was reviewed for three, six, and 12 months. On follow-up, the patient remained asymptomatic with no radiographic signs of periapical pathology, and apical closure was evident (Figure 8).

## Discussion

Non-surgical endodontic therapy, sometimes referred to as NSET, is a type of endodontic therapy that is considered to be an intrusive technique because it involves the removal of the pulp. As a result, the patient is left without regeneration, proprioception, and innervation. The condition of the pulp is of utmost importance when considered in relation to the effects of treatment. Caries are responsible for the deterioration and inflammation that take place in the pulp to varied degrees for each patient. The deterioration starts in the coronal area and subsequently advances toward the apical region of the root, and this implies the presence of vital pulp tissue in cases of mild to moderate inflammation cases. The edema prevention mechanism provides support for the compartmentalization hypothesis that is being discussed here [13]. In the region that has been subjected to aggression, the pulp’s exposure to bacteria makes the inflammation worse. It is common to see vascular events and enhanced permeability in the vicinity of the carious tissue [14].

When it comes to circumstances like IP, an approach of vital pulp therapy that is minimally invasive has been utilized. This is due to the fact that success is dependent on the healing capabilities of the remaining pulp as well as the biocompatibility of the pulp capping agents, which is a crucial component.

After the pulpotomy, it is feasible to easily determine whether or not the pulpal tissue has healed if the bleeding ceases within five to 10 minutes of the treatment [9]. In mature permanent teeth, the MTA pulpotomy has a success rate of 100% one year after the treatment has been performed [9].

To overcome the drawbacks of root canal treatment and to make use of the benefits of pulpotomy, a more promising strategy that integrates both of these procedures enables us to achieve clinical success, which is beneficial for the patient with both symptomatic IP and apical periodontitis in the long term.

In the case discussed here, the cold test was used to assess pulp sensibility since it was considered more accurate than the heat test [15]. After the removal of the temporary filling in the tooth, the mesial root of the tooth (#36) was found to be necrotic, and profuse bleeding was present in the distal root. Hence, the integrated approach was carried out. The bleeding from the distal canal was arrested by placing 5.25% sodium hypochlorite as recommended by Dammaschke et al. and Asgary et al. [16,17]. Hemostasis was achieved in three minutes, which was in accordance with the American Association of Endodontists (AAE) guidelines and was a positive sign for the success of the vital pulp therapy.

After hemostasis, MTA has been used as a pulpotomy agent since the studies have reported the success rate of MTA in permanent molars to range from 95% to 100% after a follow-up period of 12-42 months [18]. In young permanent molars, the success rate was 100% after a follow-up period of 18.9-73.6 months.

RMGIC was placed over the set MTA. Then, conventional root canal treatment was performed in the mesial canals of the tooth. The apical preparation was done till 25.6% since it is the minimum apical preparation size required for the disinfection of the canal [19].

The irrigation was done with 5 mL of 5.25% NaOCl due to its broad-spectrum antibacterial properties against species involved in endodontic infection, in addition to its ability to dissolve necrotic tissue [20]. Then, 5 mL of 17% EDTA was used as the final irrigant for one minute since EDTA is a chelating agent that can effectively remove the smear layer.

After drying, the canals were filled with premixed calcium hydroxide paste using a Lentulo spiral. The tooth was then temporarily restored with a double-seal technique as it gains the advantage of two restorative materials, i.e., the strength of one material and the sealing ability of another material [21]. In the following appointment, calcium hydroxide was removed with the help of XP Endo Finisher, and then, the canals were obturated with gutta-percha using a bioceramic sealer because of its excellent sealing ability, dimensional stability, and low solubility thereby providing a tight seal, preventing bacterial leakage and reducing the risk of reinfection [22]. On follow-up after three, six, and 12 months, the patient remained asymptomatic with no radiographic signs of periapical pathology.

Thus, it has been demonstrated that the combination of root canal therapy (RCT) and pulpotomy has a success rate of 93.3% in patients who have had the surgery [23]. “EndoVital” treatment is the term that has been given to this interventional therapeutic method [23].

To successfully perform combined pulpotomy and endodontic treatment on teeth that have been identified as having apical periodontitis, it is essential to have a correct diagnosis, maintain hemostasis throughout the pulpotomy procedure, choose the appropriate biomaterial, and have knowledge of pulp biology. This case shows that after vital pulp therapy in the distal canal, there is evidence of apical closure of the open apex, besides the absence of periapical pathology and clinical signs and symptoms, which demonstrates the clinical success of the procedure. Since only one case report has been provided here, the exact clinical outcome of this technique cannot be assessed adequately, which would be the major limitation of this article. Furthermore, more clinical studies have to be conducted in different case scenarios to exactly predict the advantages as well as disadvantages of this technique.

## Conclusions

Therefore, the integrated approach to therapy may be utilized in conjunction with an accurate diagnosis to achieve success with one strategy over another, and it is important to note that this approach is an excellent method for treating young patients, as it is minimally invasive and can also be utilized in immature teeth, which aids in root closure. On the other hand, more research and technological developments are required to optimize the treatment protocol and establish it as a standard modality that is founded on solid evidence.
